# A longitudinal study on emotional distress among local government staff seven years after the 2008 Wenchuan earthquake in China

**DOI:** 10.1186/s12889-021-10726-8

**Published:** 2021-04-09

**Authors:** Yunge Fan, Lili Guan, Hu Xiang, Xianmei Yang, Guoping Huang, Wenhong Cheng, Yongbiao Xie, Xiuzhen Wang, Guangming Liang, Ming He, Ruiru Wang, Jia Hu, Menglin Liu, Xiaojie Mou, Baoming Wu, Hong Ma, Xin Yu

**Affiliations:** 1 Peking University Sixth Hospital, Peking University Institute of Mental Health, NHC Key Laboratory of Mental Health (Peking University), National Clinical Research Center for Mental Disorders (Peking University Sixth Hospital), Haidian District Huayuan North Road No. 51, Beijing, 100191 China; 2 The Third People’s Hospital of Mianyang, Mianyang, Sichuan China; 3 Shanghai Mental Health Center, Shanghai, China; 4 Guangdong Mental Health Center, Guangzhou, Guangdong China; 5 Shenyang Mental Health Center, Shenyang, Liaoning China; 6 Hangzhou Seventh People’s Hospital, Hangzhou, Zhejiang China; 7 Peking University Third Hospital, Beijing, China; 8 Shanghai Yangpu Mental Health Center, Shanghai, China; 9 Wuhan Mental Health Center, Wuhan, Hubei China

**Keywords:** Emotional distress, Professional quality of life, Earthquake exposure, Longitudinal study, Local government staff, Disaster

## Abstract

**Background:**

The current study examined the change in local government staff’s emotional distress over 7 years after the 2008 Wenchuan earthquake, and the influence of earthquake exposure and professional quality of life (ProQOL) on emotional distress.

**Methods:**

This longitudinal study assessed 250 participants at 1 year after the earthquake; 162 (64.8%) were followed up at 7 years. Emotional distress was assessed with the Self-Reporting Questionnaire (SRQ) at both time points. We assessed ProQOL, including compassion satisfaction, burnout, and secondary traumatic stress, and earthquake exposure at 1 year. Wilcoxon signed-rank tests were performed to test longitudinal changes in emotional distress. Hierarchical multiple regression was conducted to examine the effect of earthquake exposure and ProQOL.

**Results:**

The positive screening rate of emotional distress (SRQ ≥ 8) was 37.6 and 15.4% at one and 7 years, respectively. Emotional distress scores declined over time (*p* < 0.001). Earthquake exposure and ProQOL predicted one-year (*p*s < 0.05) but not seven-year emotional distress, whereas burnout predicted both one-year (*p* = 0.018) and seven-year (*p* = 0.047) emotional distress.

**Conclusions:**

Although emotional distress can recover over time, it persists even 7 years later. Actions to reduce burnout during the early stage of post-disaster rescue have long-term benefits to staff’s psychological outcomes.

## Background

The 2008 Wenchuan earthquake, as one of the most catastrophic natural disasters in China, left hundreds of thousands of people dead or missing and millions of people homeless [1]. After the disaster, local government staff were appointed as the main workforce of post-quake relief and reconstruction owing to their extensive knowledge of the local culture and access to local resources [2].

The staff, while working as rescuers, were also survivors of the disaster. Their dual role thus exacerbated the development of psychological distress [3]. The reported prevalence of depression and anxiety among the local staff was 38.7% 1 year after the Wenchuan earthquake [4], and more than half of the staff in the extremely affected areas experienced mental disorders [5]. However, few studies have focused on the long-term psychological health of this special population [6] using a longitudinal design to investigate their mental health outcomes. As the psychological sequelae of a disaster can last for many years [7], the long-term psychological outcomes of the local government staff and the associated factors of their emotional distress are of particular interest [2].

Disaster exposure, such as bereavement and property damage, is widely known to have adverse effects on survivors’ mental health conditions [6, 8]. Previous research has documented that 12.8% of the surviving government staff who engaged in disaster relief experienced bereavement, involving loss of children, spouses, and other relatives; and more than 90% of their houses had collapsed or suffered damage [2]. Moreover, two local government staff committed suicide 11 months after the earthquake, both having lost a son in the disaster [9]. Regarding the impact of disaster exposure on long-term psychological outcomes, the existing findings are inconclusive. Shang et al. [10] reported that earthquake exposure does not significantly predict emotional distress among general survivors 3 years after a disaster, whereas Guo et al. [11] claimed that exposure to an earthquake can contribute to psychological distress at 8 years.

Engaging in the post-quake rescue and reconstruction work heavily influenced the psychological condition of local government staff [2, 3, 12]. Professional quality of life (ProQOL) is the quality of life one feels in their post-disaster rescue work. It encompasses two aspects: compassion satisfaction and compassion fatigue [13, 14]. Compassion satisfaction is the pleasure and fulfillment derived from being able to assist others and contribute to society. However, this positive aspect of being involved in post-quake rescue work remains unclear and commonly overlooked. In contrast, compassion fatigue is a common negative consequence when working with trauma as rescuers [14–16], and comprises of burnout and secondary traumatic stress [13]. It is generally found that compassion fatigue is associated with negative psychological outcomes [17–25]. Regarding the longitudinal influence of ProQOL on mental health, three studies conducted among relief workers after the Great East Japan Earthquake have revealed the effect of cumulative burnout on psychiatric distress at 2 to 4 years using a cross-sectional design [23–25]. Nevertheless, caution must be exercised when interpreting and generalizing the findings owing to the dearth of longitudinal studies [18, 26].

Indeed, the existing literature has several limitations. Firstly, although the local government staff are crucial manpower during post-quake relief and reconstruction, the psychological consequences to this special population tend to receive minimal research attention [6]. Secondly, previous studies on mental health of local government staff have been conducted within the first year after the earthquake [2, 4, 5, 9, 12], and were cross-sectional in design [6, 18, 26]. As the reconstruction following natural disasters, such as earthquakes, typically lasts for 10 years [27, 28], the long-term psychological consequence among the staff needs to be examined. There is a general lack of longitudinal research on the effects of emotional distress after a disaster. Thirdly, earthquake exposure and ProQOL were recognized as important factors contributing to the psychological status among general survivors and rescuers, respectively. However, their impact on the long-term emotional conditions among the local government staff, who were also rescuers after the disaster, remains unclear.

In light of the past studies and their limitations, the present longitudinal study aimed to describe the changes in emotional distress among local government staff over 7 years after the Wenchuan earthquake. The effects of earthquake exposure and ProQOL on the staff’s long-term mental health were also investigated.

## Methods

### Participants

A total of 250 participants were recruited from two badly hit disaster areas, namely, counties of Beichuan and An. Eligible participants were those who experienced the earthquake and local government staff engaging in the post-disaster relief and reconstruction. The first assessment was conducted in September 2009, approximately 1 year after the earthquake. The follow-up assessment was in December 2015, 7 years after the earthquake; 162 (64.8%) participants completed the second assessment at 7 years. Among the 162 available participants at 7 years, 136 were still government staff and 26 were retired. Eighty-eight participants were not available at 7 years because of death (*n* = 2), refusal (*n* = 3), retirement (*n* = 41), exit from government service (*n* = 33), or relocation (*n* = 9) based on the report of the local county government. All the eligible government staff from the two counties were approached for the one-year assessment. Two hundred and fifty participants from 240 different villages were available and willing to participate in the study, representing 44% of the whole eligible staff at that time. With 250 participants at 1 year and 162 at 7 years, the power to detect a small (Cohen’s *f*^*2*^ = 0.02), median (*f*^*2*^ = 0.15), or large (*f*^*2*^ = 0.35) effect was 0.605, 0.999, or 1.000 at 1 year, and 0.432, 0.998, or 1.000 at 7 years, respectively. The study was approved by the Ethics Committee of Peking University Sixth Hospital, with written informed consent obtained from all participants.

### Procedures

To approach the potential participants, we first introduced details of the study to the local county governments and secured their approval and collaboration. Next, we contacted all local government staff working at the counties one by one. The staff were recruited if they agreed to participate in the study. During on-site interviews, they were instructed to complete a packet of measures to assess their emotional distress, earthquake exposure, and ProQOL at 1 year. All participants recruited at 1 year after the earthquake were informed that they might be contacted for follow-up interview on a voluntary basis. The research team contacted them again for the seven-year assessment, during which the measure of emotional distress was reassessed.

### Measures

#### Emotional distress

The Self-Reporting Questionnaire (SRQ) was used to assess the emotional distress of participants for the one- and seven-year assessments. It was developed by the World Health Organization (WHO) as a reliable and valid instrument designed to screen for depression, anxiety, somatic-related symptoms, and other symptoms [29–32]. The Chinese version of the SRQ interview guide has previously been published [33] and demonstrated good reliability and validity [33, 34]. The permission for use of SRQ in this study was granted by WHO. The SRQ consists of 20 questions that have to be answered using yes or no. Each question was scored “0” or “1” to indicate that the symptom was absent or present, respectively, during the past month. Sum scores were used in the analysis, with the maximum score being 20. Higher scores indicate increasingly severe emotional distress. A score of 7 or 8 is recommended to be used as the cut-off to detect psychiatric disorders [30, 33, 35]. The present study used SRQ score ≥ 8 to identify persons with positive screenings of emotional distress. The positive screening rates for the one- and seven-year assessments were calculated.

#### Earthquake exposure

Earthquake exposure was assessed with four self-designed items at the first time point (1 year after the earthquake). The first was house damage, for which three options were provided: “no,” “moderate,” and “severe,” recorded as “0,” “1,” and “2,” respectively. Moderate referred to the case where the house was partially damaged but habitable, whereas severe was defined as the total collapse of the house. The second item was the number of moves after the earthquake. The government established many temporary shelters after the earthquake. Survivors could leave the temporary shelters and return to their own house when their houses had been repaired and the public health emergency had stabilized. Some staff as survivors might have needed to move several times among different temporary shelters, as temporary communities were combined when a certain number of survivors returned home. A larger number of moves indicates higher earthquake exposure risk [36]. The third item was the number of relatives who died. Participants were required to report how many of their relatives died in the earthquake. In the last item, injury, participants were asked to indicate if they were injured in the earthquake. Responses were recorded as “0” (no) or “1” (yes).

#### ProQOL

Professional quality of life was assessed by the Professional Quality of Life Scale (ProQOL) at the one-year assessment. The ProQOL is a self-reported scale developed specifically for rescuers working with individuals who have experienced extremely stressful events, with demonstrated validity and reliability [14, 37]. The Chinese version of ProQOL has previously been published [38, 39] and has shown good psychometrics properties among local government staff after the Wenchuan earthquake [38]. In the current study, ProQOL is applied to indicate the quality of life local government staff perceived when they were engaging in the post-quake relief and reconstruction as rescuers.

The use of ProQOL was permitted by the ProQOL Office. It contains 30 items with three subscales: compassion satisfaction, burnout, and secondary trauma stress. Participants were instructed to answer how frequently they had experienced each situation when they were helping other survivors or engaging in the post-quake relief and reconstruction work in the last month. Each item is scored on a five-point Likert scale (1 = never, 2 = rarely, 3 = sometimes, 4 = often, 5 = very often). Scores were calculated by separately summing items in the three subscales, with possible scores ranging from 10 to 50 for each subscale. Higher scores represent higher levels of compassion satisfaction, burnout, or secondary trauma stress [14, 40].

### Statistical analyses

For the longitudinal changes in emotional distress, Wilcoxon signed-rank tests were performed. Hierarchical multiple regression analyses were conducted to examine whether earthquake exposure and ProQOL, separately, would predict emotional distress at 1 and 7 years after the earthquake. All models were adjusted for demographic covariates, including age, sex, education, religion, marital status, and family income, which were entered as a block in the first step, followed by the earthquake exposure or ProQOL variables (compassion satisfaction, burnout, and secondary trauma stress) in the next step.

As 64.8% participants were retained at 7 years, we meant to examine whether the dropouts were selective which may induce bias. Chi-squared and independent sample *t* or Mann–Whitney *U* tests (if data failed the assumption of normal distribution) were used to compare between the followed-up group and drop-out group, in terms of demographics, earthquake exposure, ProQOL, and emotional distress assessed at 1 year.

## Results

### Participant characteristics

The demographic characteristics, earthquake exposure, ProQOL, and emotional distress of all participants are presented in Table 1. Eighty-eight participants were unavailable for the follow-up assessment at 7 years after the earthquake. Compared to the participants followed at 7 years, those who dropped out were more likely to be somewhat older. Notably, the dropout group included a larger proportion of retired staff. Apart from age, no significant differences were found between the participants surveyed at 7 years post-quake and those dropouts in any of the other demographic variables, earthquake exposure, ProQOL, and emotional distress scores. The 162 available participants at the seven-year assessment were responsible for a total of 219,753 survivors according to their reports.

**Table 1:** Descriptive statistics of participants’ demographic characteristics, earthquake exposure, professional quality of life, and emotional distress assessments

Variables	1 year (***N*** = 250)	7 years (***n*** = 162)	7-year dropouts (***n*** = 88)	Test of difference ^**a**^
Demographic				
Age, mean (SD) years	47.06 (7.25)	45.87 (6.41)	49.25 (8.20)	*t* = − 3.35^**^
Male	239 (95.6%)	153 (94.4%)	86 (97.7%)	*χ*^*2*^ = 1.46
Education years, mean (SD)	10.57 (2.46)	10.76 (2.31)	10.23 (2.70)	*t* = 1.64
Without religion	238 (95.2%)	153 (94.4%)	85 (96.6%)	*χ*^*2*^ = 0.58
Married or partnered	245 (98.0%)	159 (98.1%)	86 (97.7%)	*χ*^*2*^ = 0.05
Family income, mean (SD) RMB	1866.22 (1647.69)	1860.38 (1785.59)	1876.90 (1369.84)	*z* = − 0.01
Earthquake exposure				
House damage				*χ*^*2*^ = 0.88
No	10 (4.0%)	8 (4.9%)	2 (2.3%)	
Moderate	86 (34.4%)	57 (35.2%)	29 (33.0%)	
Severe	148 (59.2%)	97 (59.9%)	51 (58.0%)	
Number of moves after earthquake				*χ*^*2*^ = 5.89
0	80 (32.0%)	53 (32.7%)	27 (30.7%)	
1	53 (21.2%)	29 (17.9%)	24 (27.3%)	
2	49 (19.6%)	34 (21.0%)	15 (17.0%)	
3	38 (15.2%)	24 (14.8%)	14 (15.9%)	
4	19 (7.6%)	13 (8.0%)	6 (6.8%)	
5	9 (3.6%)	8 (4.9%)	1 (1.1%)	
6	1 (0.4%)	1 (0.6%)	0	
Number of relatives died				*χ*^*2*^ = 4.72
0	217 (86.8%)	141 (87.0%)	76 (86.4%)	
1	19 (7.6%)	14 (8.6%)	5 (5.7%)	
2	7 (2.8%)	3 (1.9%)	4 (4.5%)	
3	3 (1.2%)	1 (0.6%)	2 (2.3%)	
4	1 (0.4%)	1 (0.6%)	0	
5	0	0	0	
6	2 (0.8%)	1 (0.6%)	1 (1.1%)	
7	1 (0.4%)	1 (0.6%)	0	
Injured	17 (6.8%)	8 (4.9%)	9 (10.2%)	*χ*^*2*^ = 2.52
Professional quality of life ^b^ at 1 year				
Compassion satisfaction score, mean (SD)	39.33 (4.84)	39.08 (4.82)	39.81 (4.88)	*t* = −1.12
Burnout score, mean (SD)	24.07 (4.68)	24.36 (4.45)	23.51 (5.07)	*t* = 1.34
Secondary traumatic stress score, mean (SD)	24.88 (5.87)	24.84 (5.78)	24.96 (6.07)	*t* = −0.16
Emotional distress ^c^ at 1 year, mean (SD)	6.31 (4.21)	6.49 (4.27)	5.98 (4.09)	*z* = −0.84

^a^Test of difference between available participants and those dropouts at 7 years

^b^Professional quality of life was measured by the Professional Quality of Life Scale, which includes compassion satisfaction, burnout, and secondary traumatic stress subscales. Higher scores indicated higher levels of compassion satisfaction, or higher risks for burnout or secondary traumatic stress

^c^Emotional distress was measured by the Self-Reporting Questionnaire, with higher scores indicating more severe emotional distress

**p < 0.01

### Emotional distress change

**Fig. 1:**
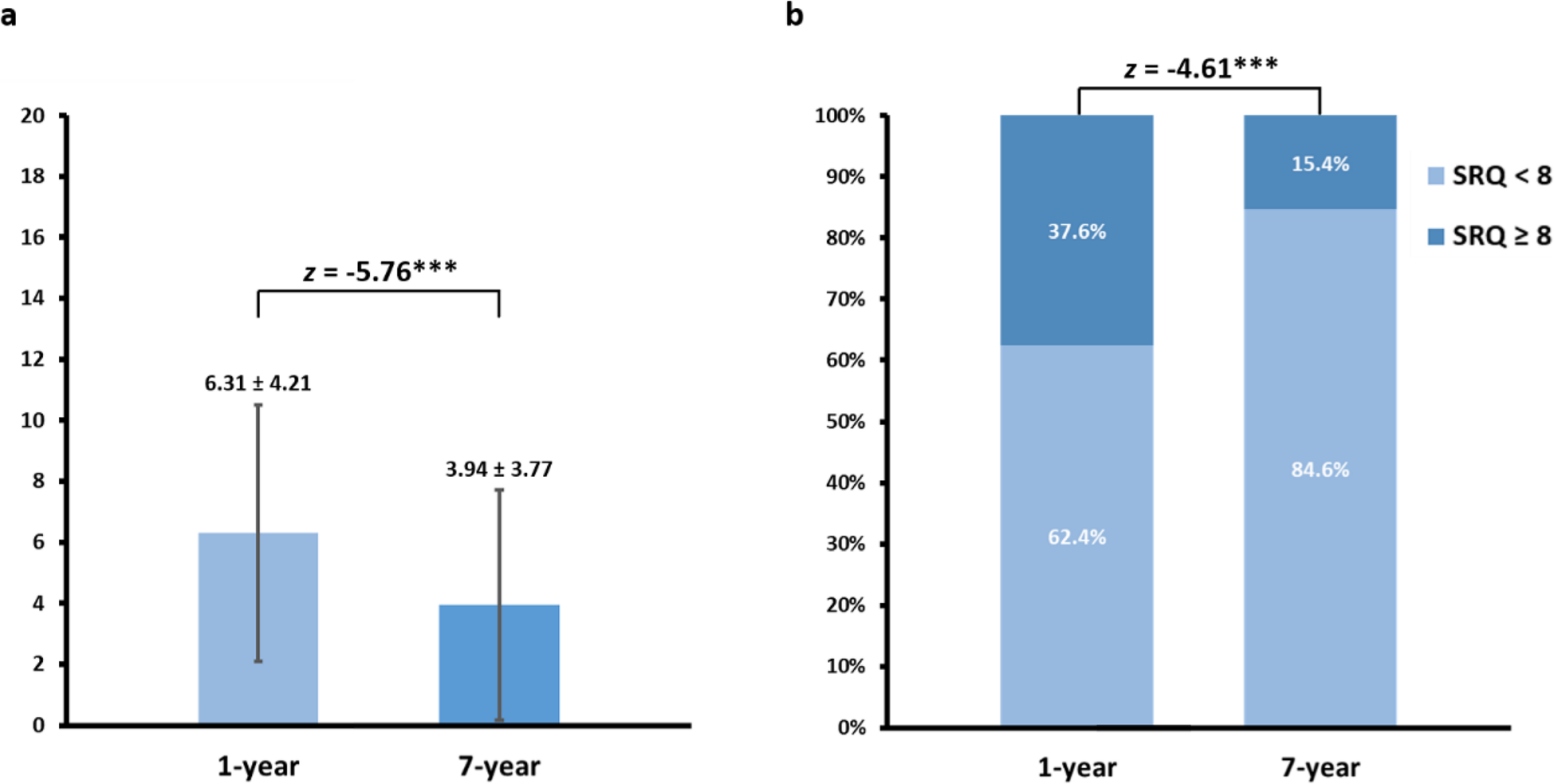
Emotional Distress at 1 and 7 Years after the Wenchuan Earthquake*.* (a) Continuous scores of emotional distress at each measurement time point. Emotional distress was measured by the Self-Reporting Questionnaire (SRQ), with higher scores indicating more severe emotional distress. (b) Percentages of SRQ score ≥ 8 and SRQ score < 8 at each measurement time point. Statistics *z* represent differences between the two assessments. Standard deviations are represented by vertical bars. *** *p* < 0.001

The continuous SRQ scores were 6.31 (SD = 4.21) and 3.94 (SD = 3.77) at 1 and 7 years, respectively. The percentages of SRQ score ≥ 8 were 37.6 and 15.4% at 1 and 7 years, respectively. As shown in Fig. 1, both the continuous SRQ scores (*z* = − 5.76, *p* < 0.001) and the percentages of SRQ score ≥ 8 (*z* = − 4.61, *p* < 0.001) declined significantly from 1 year to 7 years for the participants as a whole. In addition, the SRQ scores at the two assessment time points showed a significant correlation (*r* = 0.23, *p* = 0.003).

### Earthquake exposure in prediction of emotional distress at 1 and 7 years

Results from the regression models of earthquake exposure predicting emotional distress are shown in Table 2. Tests of the assumption of collinearity indicated that multicollinearity was not a concern (variance inflation factors, VIFs = 1.02 to 1.39). After adjusting for demographic covariates, earthquake exposure was found to predict one-year emotional distress significantly (*∆R*^*2*^ = 0.048, *p* = 0.021) but did not account for the seven-year emotional distress (*∆R*^*2*^ = 0.012, *p* = 0.762).

**Table 2:** Regression analyses of earthquake exposure and professional quality of life predicting emotional distress at 1 and 7 years after the Wenchuan earthquake

Predictors	Prediction of one-year emotional distress ^**a**^			Prediction of seven-year emotional distress ^**a**^		
	***β***	***p***	***∆R***^***2***^	***β***	***p***	***∆R***^***2***^
Model 1 ^b^: Earthquake exposure			0.048^*^			0.012
House damage	−0.02	0.793		−0.01	0.921	
Number of moves after earthquake	0.17	0.011		0.01	0.982	
Number of relatives died	0.14	0.036		−0.03	0.764	
Injured	−0.05	0.464		−0.11	0.211	
Model 2 ^b^: Professional quality of life			0.250^***^			0.032
Compassion satisfaction score	−0.11	0.103		0.04	0.680	
Burnout score	0.17	0.018		0.21	0.047	
Secondary traumatic stress score	0.39	< 0.001		−0.03	0.745	

^a^All models adjusted for age, sex, education, religion, marital status, and family income

^b^Models 1 and 2 were tested separately

* *p* < 0.05, *** *p* < 0.001

### ProQOL in prediction of emotional distress at 1 and 7 years

For the models of ProQOL (compassion satisfaction, burnout, and secondary trauma stress) predicting emotional distress (as shown in Table 2), no multicollinearity was presented (VIFs = 1.34 to 1.67). ProQOL was significantly associated with one-year emotional distress (*∆R*^*2*^ = 0.250, *p* < 0.001) while controlling for demographic covariates. In the prediction of seven-year emotional distress, ProQOL, in total, non-significantly explained the variance (*∆R*^*2*^ = 0.032, *p* = 0.167). To be more specific, higher levels of burnout (*β* = 0.17, *p* = 0.018) and secondary trauma stress (*β* = 0.39, *p* < 0.001) experienced by the staff at 1 year after the earthquake significantly predicted more severe emotional distress at the same time point, while controlling for demographic covariates. In prediction of seven-year emotional distress, the effect of one-year burnout was still significant (*β* = 0.21, *p* = 0.047), while compassion satisfaction (*β* = 0.04, *p* = 0.680) and secondary trauma stress was not (*β* = − 0.03, *p* = 0.745).

## Discussion

Local government staff, who were both survivors and key rescuers after a disaster, represent an extremely important but under-studied population globally. To the best of our knowledge, this study is the first to investigate the long-term changes in emotional distress among local government staff and the long-term effect of disaster exposure and rescue experience on their emotional distress using a longitudinal study design. The participants in this study served more than 220,000 general survivors.

The results that SRQ scores declined significantly from 1 to 7 years indicated that the staff’s emotional distress recovered over 7 years after the earthquake. Few existing studies were found to investigate emotional outcomes among the staff population after a disaster and assess their long-term mental health wellbeing. Considering that the staff participants recruited in this study were not only rescuers but also survivors, it might be of interests to compare with other studies including general survivors or rescuers after the earthquake. After Wenchuan earthquake, the positive screening rate of distress among the local government staff (37.6% at 1 year and 15.4% at 7 years) was found to be slightly lower compared with general survivors at both time points [11, 41–45], but largely higher compared with other rescuer populations [46–48]. The estimated prevalence of emotional distress was demonstrated to be 26% among medical rescue workers at 1 year [46], and less than 5% among both rescue soldiers and health care workers 2 years after the Wenchuan earthquake [47, 48]. Consistent with previous studies of general survivors or rescuers [11, 47], the present study indicated that both the positive screening rate and severity of emotional distress declined over time in our sample. Nonetheless, there was one in every six to seven participants in our sample who continued to present with mental health issues even 7 years after the earthquake. These findings imply that the long-term development of emotional well-being among local government staff needs to be taken seriously during the extended duration of post-quake rescue and reconstruction. Timely and sustainable psychosocial support, such as emotional support from love ones and professionals, and financial compensation, is of particular importance to government workers for their long-term mental health and functioning [43, 49].

Meaningful engagement, such as the pleasure when assisting others and feelings of fulfillment when contributing to society, could alleviate staff’s emotional distress [12, 14, 16, 50]. However, the findings of our analysis did not find any significant contributory effect of compassion satisfaction, the positive aspect of ProQOL. The measure of compassion satisfaction assessed generic satisfaction when helping others, and as such, it might not be sufficient to capture the full range of positive experience of post-quake rescue, limiting its predictive power. Further research is needed to explore more specific protective factors of rescue work, such as confidence, sense of optimism, and feelings of fulfillment. Subsequently, interventions to promote meaningful and beneficial engagement in rescuers need to be developed. Another plausible reason might be that the effect of compassion satisfaction is not large enough to be detected with the current sample size. Future studies may investigate positive impact of post-disaster rescue experience with a larger sample size.

This study also examined the long-term impact of compassion fatigue in a disaster context. Although burnout and secondary trauma stress are both identified as compassion fatigue, burnout is a state of physical, emotional, and mental exhaustion that is caused by prolonged involvement in emotionally demanding situations [51], while secondary traumatic stress is defined as a traumatic response that individuals might develop when helping trauma survivors. This is about work-related, secondary exposure to people who have experienced extremely traumatic events [13]. Burnout tends to result from working overload and overtime [19, 52], whereas secondary trauma stress relates to susceptibility to others’ trauma experience [14]. According to our results, the influence of burnout on emotional distress lasted throughout the seven-year duration of the study. In contrast, the impact of secondary trauma stress on emotional distress diminished among the majority of staff, which may be due to the psychological and physical recovery of general survivors over time. To address the issue, methods to effectively decrease burnout among local government staff, such as lessening workload and avoiding extended working hours, providing psychosocial interventions, and delivering professional capacity training, should be implemented in a timely and continuous manner during the long-term post-disaster relief and reconstruction progress [19, 52–54]. However, although it was not systematically measured in this study, continuous occupational and psychological intervention was rarely conducted among the staff population after the earthquake, according to some of participants’ reports. Additionally, secondary trauma stress should not be overlooked; according to our analysis, it had a strong effect in predicting short-term distress and lasted among part of the high-risk staff at 7 years. Interventions addressing staff traumatic stress should be implemented immediately after a disaster. Continuous follow-up of staff with higher risks of traumatic experience for a long period may also be warranted.

Earthquake exposure has been recognized as a risk factor for mental health issues within the first year following a disaster [6], while no association was found between earthquake exposure and emotional distress at 7 years after the disaster. This might be because the earthquake exposure measured in this study related to objective phenomenon, such as being injured, suffering bereavement, and witnessing injury or death. As shown in literature, subjective earthquake exposure measures, such as feelings of fear being injured or killed, are longitudinally associated with general psychological distress [10, 11]. Further research could include assessments of both subjective and objective earthquake measures to elucidate fully the impact of disaster exposure on the progression of long-term mental health issues.

The authors are aware of the limitations of the present study. Firstly, although this longitudinal study provides evidence on the trends of change in local staff’s emotional distress from the earlier to later stages after a natural disaster, the time interval between the two assessments is relatively large. Future studies should track the staff more frequently over multiple time points to observe the developmental trajectories of their mental health status over time. Secondly, the SRQ used in this study is a screening instrument rather than a clinical diagnostic method. Thus, the positive screening rate of emotional distress may be considered with caution. Future research should implement a clinical diagnostic instrument when possible. Thirdly, as subjective measures of disaster exposure tend to be more important predictors of long-term psychological distress, further research could pay more attention to subjective disaster exposure and its impact on the progression of lasting mental health issues.

The present findings contribute to the understanding of the long-term consequences of natural disasters, particularly in the field of mental health. As for clinical implications, our study suggests that health professionals should monitor the psychological distress of local government staff consistently during the entire post-quake relief and reconstruction progress, and subsequently deliver timely intervention to prevent deterioration and facilitate recovery. In addition, burnout tends to have lasting detrimental effects on staff distress. Effective solutions to reduce burnout, such as lessening the staff’s workload, avoiding extended working hours, carrying out shift-work schedules, providing continuous psychosocial interventions, and delivering professional capacity training, may be beneficial to improve future long-term mental health outcomes of local government staff and rescue workers after major disasters. Moreover, assessment and intervention addressing staff’s secondary trauma stress should be implemented immediately after a disaster.

## Conclusions

Despite emotional distress being liable to recover over time, it continues to persist among local government staff even 7 years after the Wenchuan earthquake. The findings underscore the importance of taking action to reduce burnout in local staff during the early stage of post-disaster rescue work, which would be salutary to their psychological outcomes in the long run.

## Data Availability

The dataset used and/or analyzed during the current study is available from the corresponding author on reasonable request.

## Abbreviations

ProQOL: Professional quality of life
SRQ: Self-Reporting Questionnaire
